# How the Big Five personality traits related to aggression from perspectives of the benign and malicious envy

**DOI:** 10.1186/s40359-022-00906-5

**Published:** 2022-08-18

**Authors:** Xinsheng Jiang, Xiaojun Li, Xia Dong, Lan Wang

**Affiliations:** 1 School of Educational Science, Hunan Normal University, Changsha, China; 2 School of Teacher Education, Nanjing Xiao Zhuang University, Nanjing, China; 3 School of Social and Behavioral Science, Nanjing University, Nanjing, 210023 China

**Keywords:** Big Five personality traits, Aggression, Benign/malicious envy

## Abstract

**Background:**

Prior studies have indicated the link between the Big Five personality traits and aggression. Based on the general aggression model, the purpose of this study is to reveal the formation mechanism of aggression from the people’s internal emotional perspective. Envy is a typical negative emotion that can be divided into benign/malicious envy. Therefore, we aim to explore the intrinsic role of benign/malicious envy within the Big Five personality traits in its connection to aggression.

**Methods:**

We recruited 839 participants [229 men (27.29%) and 610 women (72.71%); mean age ± *SD* = 19.45 ± 2.39] who we tested with the NEO Personality Inventory, the Benign and Malicious Envy Scale, and the Aggression Questionnaire.

**Results:**

The results of suggested that neuroticism was significantly and positively associated with aggression, while agreeableness was negatively related to aggression. Moreover, mediation analysis revealed that malicious envy works both in the relationship of neuroticism-aggression and agreeableness-aggression.

**Conclusions:**

The current study advanced knowledge of the general aggression model. Most importantly, it reveals that malicious envy, as a type of envy, plays an important mediating role between neuroticism, agreeableness and aggression. Meanwhile, the cross-gender path analysis supports the stability of the mediating role of malicious envy. This finding provides new insights into the intervention of aggression from the perspective of envy.

## Introduction

Aggression can be defined as actions that intentionally inflict harm on others [1], and including the four sub-traits of physical aggression, verbal aggression, anger, and hostility [2]. It is very common in society. Aggression manifests itself in the form of war, terrorism, and assassination [3]. Based on the general aggression model (GAM), person and situation factors trigger internal, emotional, and cognitive routes that make people engage in aggressive behavior [1]. The link between aggression and personality, as affecting factors, has attracted much attention. Numerous studies have explored the connection between the Big Five personality traits and aggression [4–6]. The current study focuses on emotional perspective, especially benign/malicious envy in order to bring to light the relationship between Big Five personality traits and aggression. Envy is a basic negative emotion [7]. It can also be divided into two completely different forms: benign and malicious [8]. Do people with different personality traits display aggression differently, related by benign/malicious envy? Based on the GAM, the purpose of this study is to reveal the intrinsic role of benign/malicious envy in the big Five personality traits and aggression, and measure all variables at the trait level, especially aggression and envy.

### Big Five personality traits and aggression

The Big Five personality traits include five facets: neuroticism, extraversion, openness to experience, agreeableness, and conscientiousness [9, 10]. Neuroticism refers specifically to people’s tendency to be anxious, hostile, and impulsive. Extraversion reflects people’s tendency to be enthusiastic, optimistic, and social. Openness to experience reveals people’s tendency to innovate and seek out different solutions. Conscientiousness manifests itself in the tendency to have due diligence and self-discipline Agreeableness reflects people’s level of trust, altruism, and straight-forwardness [9].

Which Big Five personality traits are associated with aggression? Anderson and Bushman [1] proposed the GAM, which contains three routes: inputs of person and situation; internal emotional, and cognitive processes; and assessment and decision-making. Based on the GAM, people with different personality traits may have different emotional and cognitive routes when facing the same situation [1]. As a result, some people may show aggressive impulses and behaviors, while others may not [11, 12]. For instance, people with higher levels of neuroticism are more likely to experience painful and irrational thoughts. They may then engage in aggressive behaviors [11, 13]. On the other hand, people with higher levels of agreeableness are less likely to engage in aggressive behaviors [14].

Meanwhile, prior studies have explored the relationship between the Big Five personality traits and situational/dispositional aggression separately. Regarding situational aggression, Bettencourt et al. [15] used a scenario-induced method. They found that neuroticism positively correlated with aggression, whereas agreeableness negatively linked with aggression. A large amount of research on dispositional aggression has also been conducted. Dam et al. [4] revealed that agreeableness and conscientiousness were negatively associated with aggression, whereas neuroticism was positively related to aggression. Li et al. [16] found that neuroticism positively linked with aggression, whereas agreeableness negatively correlated with aggression. Gleason et al. [17] indicated that openness to experience tends to be unlinked with aggression. However, the relationship between extraversion and aggression is mixed. Sharpe and Desai [18] found that the relationship between extraversion and aggression was negative, whereas Gallo and Smith [19] revealed a positive link between extraversion and aggression. In conclusion, the link the Big Five personality traits and dispositional aggression had slight complex, whereas the relationship between neuroticism, agreeableness and aggression remains relatively stable. In this regard, we expect the present study to corroborate this pattern of interrelation, and also explore the relationship between openness to experience, extraversion, conscientiousness and aggression.

### The mediating role of benign/malicious envy

Envy that comes from an adverse upward social comparison is an unpleasant emotion that arises when we are at a disadvantage compared to others [20]. Some studies determined that envy is a painful irrational emotion [21, 22]. Alternatively, envy can be divided into benign and malicious according to other findings [8]. Prior studies have found both benign and malicious envy at the state level [23–25]. Lange and Crusius [26] research further revealed that similar to the state level, there are two distinct types of envy at the trait level: dispositional benign envy and malicious envy. The dual envy theory proposes that these two different forms of dispositional envy are derived from a deep-rooted sense of inferiority, a persistent tendency to compare and the painful experience of comparing with upward [24, 26, 27]. This research focuses on the two types of envy at the trait level.

Furthermore, appraisal theory holds that each emotion is associated with a specific appraisal model, which is the cognition of the perceptual antecedents of emotional experience [28]. Accordingly, dispositional benign/malicious envy also have their own appraisal patterns [27, 29, 30]. This difference is mainly reflected in deservingness and control potential [8, 30]. Meanwhile, these two distinct forms of envy have different emotional experiences [27, 31]. Specifically, comparison with undeserving people and the self-evaluation pattern of low control potential will trigger malicious envy, and thus more likely to experience depression and hostility [30, 31]. On the contrary, comparison with people who deserve an advantage and the self-evaluation pattern of high control potential will lead to benign envy and thus less likely to experience depression and dejection [30, 32]. Meanwhile, the aim of the malicious envy is to put down the envied person and remove them from their superior position [26, 27, 33]. Benign envy is also a frustrating experience, but its motivation is to improve oneself [23, 30, 34]. Therefore, benign envy has a positive side [35, 36].

According to the GAM, this study focuses on emotional perspective [1], mainly the emotional characteristics of envy. Namely, people with different personality traits may experience various levels of negative emotions, and thus have different levels of relationship with aggression. Based on the above, we would like to explore the role of these two types of envy within the Big Five personality traits and its link to aggression.

We will first consider the link between neuroticism and benign/malicious envy. Few studies have directly explored the relationship between the two, Smith et al. [37] found a positive correlation between neuroticism and dispositional envy. The established coefficient equaled 0.41–0.56. It is important to stress that the above studies referred to envy as a malicious emotion that contains hostile elements. In this case, people with higher levels of neuroticism may be more likely to experience malicious envy. Compared to malicious envy, benign envy has a number of beneficial aspects and allows for more positive regard of its object [26, 27, 35]. Thus, neuroticism may be negatively correlated with benign envy. Neuroticism individuals may produce more hostile and painful negative emotions [9, 38]. Individuals generally tend to make upward social comparisons, and if they perceive their status as threatened, neuroticism individuals are more likely to experience malicious envy [39, 40], which may be associated with aggression [41].

Additionally, agreeableness is the tendency to be compassionate and cooperative to others, and to care about social harmony [42]. People with higher levels of agreeableness are less likely to experience a feeling of depression [43, 44], hostility and resentment [45]. Agreeableness individuals are usually considerate, friendly, generous, helpful and willing to compromise their own interests with others [42]. Thus, individuals generally tend to make upward social comparisons, agreeable individuals may experience the benign kind of envy when they perceive themselves as disadvantaged, lowering their chances to engage in aggressive behaviors. Therefore, benign/malicious envy may be the underlying connection between agreeableness and aggression.

As far as the link between other personality (conscientiousness, openness to experience, extraversion) and benign/malicious envy. Conscientiousness features are to show self-discipline, act dutifully [9]; openness to experience is characterized by curious, good at appreciating art, and sensitive to beauty [10]. People with higher levels of extraversion enjoy being with people, they are often enthusiastic [9, 10]. Positive emotions are one of the characteristics of extroversion [9]. Considering that there is no research to explore the relationship between these three personalities and benign/malicious envy, this research is only a preliminary speculation on the relationship between them. Future research should further to explore the relationship between these three personality traits and benign/malicious envy.

Interestingly, a number of research papers have examined the link between benign/malicious envy and aggression incidentally. Smith et al. [37] provided supportive evidence in favor of the hypothesis that the sense of injustice and envy may result in aggression. Smith and Kim [8] argued that envy might lead to aggression because of the feeling of frustration. Moreover, Bao-Pei and Lei [46] found that envy can lead to multiple types of aggression, such as interpersonal conflicts and vicious crimes. Additionally, envy has been considered a sin for a long time [25]. It also appeared to be a hostile emotion that often prompts aggressive behaviors [8]. In the above studies, envy is often referred to as malicious. As a result, people with a higher degree of malicious envy are more likely to engage in aggressive behaviors. In contrast, people with higher benign envy are tend to experience less hostility, resentment, and dejection [23, 25, 30, 36], thus these subjects are less likely to engage in aggressive behaviors.

### The current study

Based on the GAM, this article intends to explore the relationship between the Big Five personality traits and aggression. We further explore the mediating role of different forms of envy. Considering that the link between neuroticism, agreeableness and aggression remains relatively stable. Therefore, benign/malicious envy may be a promising mediating role that connects neuroticism, agreeableness and aggression. Furthermore, Kahlbaugh and Huffman [47] found that conscientiousness, openness to experience and extraversion were positively related to positive emotions. This shows that these three personalities are positively associated with positive emotions, while envy is negative emotions. Therefore, these three personalities may be negatively correlated with envy. However, benign envy has a positive side [35, 36]. Based on this, conscientiousness, openness to experience and extraversion also may be negatively correlated with malicious envy and positively related to benign envy. However, there are uncertainties and ambiguities in the relationship between other personality traits (conscientiousness, openness to extraversion, experience) and aggressiveness, and it is difficult to make clear hypotheses in this paper. Based on this, we propose the following hypotheses:

#### Hypothesis 1

Neuroticism would positively correlate with aggression; agreeableness would negatively correlate with aggression.

#### Hypothesis 2

Neuroticism would be positively correlated with aggression through associating malicious/benign envy; agreeableness would be negatively correlated with aggression through associating benign/malicious envy.

## Methods

### Participants and procedures

The current study recruited 866 participants from four universities (i.e., Hunan Normal University, South China Normal University, Huazhong University of Science and Technology) in central and southern China, all of which are key national public universities. According to previous studies, there are two criteria for screening participants [35, 48]. First, a survey was excluded if more than 2/3 of the questions were blank. Second, a questionnaire was excluded if all the questions had the same answer, as this indicated that the participant did not answer them carefully. Based on this, we excluded 27 questionnaires. The final sample included 839 participants, including 229 men (27.29%) and 610 women (72.71%), with an average age of 19.45 ± 2.39. Meanwhile, the age range of participants was between 17 and 26.

Participants completed a series of questionnaires, including the NEO Personality Inventory, the Benign and Malicious Envy Scale and the Aggression Questionnaire. It took them about 40 min to fill out these questionnaires. It should be noted that the data for this study were from an ongoing project named "Philosophy and Social Science Project of Hunan Province of China (18YBA324)", some of the data have been used in previous studies [35, 36, 48]. After completing the questionnaire, all the participants were paid 30 yuan. In the meantime, all the participants provided written informed consent to participate in the study. Ethics approval for this study and protocol was obtained from the Academic Committee of the School of Psychology of Hunan Normal University.

### Measures

#### NEO personality inventory

The revised questionnaire of the Five-Factor model of personality with 120 items was used [9]. Five facets were measured, as follows: neuroticism (e.g., I rarely overindulge in anything.), extraversion (e.g., I sometimes fail to assert myself as much as I should), openness to experience (e.g., Aesthetic and artistic concerns aren’t very important to me.), agreeableness (e.g., I’m hard-headed and tough-minded in my attitudes.), and conscientiousness (e.g., I’m known for my prudence and common sense.). All items were rated by using a 5-point Likert scale and measured from 1 = strongly disagree to 5 = strongly agree. Previous studies demonstrated the reliability of this questionnaire for the Chinese sample [49]. In this study, the Cronbach alpha coefficient of the five dimensions was neuroticism: 0.80; extraversion: 0.77; openness to experience: 0.70; agreeableness: 0.69; and conscientiousness: 0.72.

#### Benign and malicious envy scale

The benign and malicious envy scale included 10 items accompanied by the 6-point Likert scale, ranging from 1 (strongly disagree) to 6 (strongly agree). Each scale consisted of five items that were developed by Lange and Crusius [26]. Benign envy items were listed, such as “If someone has superior qualities, achievements, or possessions, I try to attain them for myself.” Malicious envy items were equally presented, such as “If other people have something that I want for myself, I wish to take it away from them.” Dong et al. [35] demonstrated good reliability for the Chinese sample. In the present study, they also showed adequate reliability. Specifically, Cronbach’s alpha was respectively 0.81 and 0.85 for the benign envy scale and malicious envy scale. Moreover, confirmatory factor analysis on Chinese Benign and Malicious Envy Scale by SEM (structural equation modeling), and the results show that the model fits well (*χ*^2^ = 92.13, *df* = 31; *p* < 0.001; SRMR = 0.04, RMSEA = 0.05, CFI = 0.98, GFI = 0.98). Therefore, it conformed to the two-factor structure.

#### Aggression questionnaire

An aggression scale of 29 items was developed by Buss and Perry [2]. This scale included the following four subscales: physical aggression (e.g., If somebody hits me, I hit back.), verbal aggression (e.g., I often find myself disagreeing with people.), anger (e.g., Some of my friends think I’m a hothead.), and hostility (e.g., Other people always seem to get the breaks.). All the items were rated using the 5-point Likert scale and measured from 1 = strongly disagree to 5 = strongly agree. Xiang et al. [50] showed the validity of the questionnaire among Chinese groups. The Cronbach alpha coefficient for all 29 items was 0.89.

### Statistical analysis

We firstly made a bivariate association with all the variables in the NEO Personality Inventory, the Benign and Malicious Envy, and the Aggression Questionnaire. Second, we used structural equation modeling to analyze the relationship between Big Five personality and aggression. To be specific, we used a path analysis to further explore the relationship between Big Five personality, aggression and benign/malicious envy. Multiple indicators were used to evaluate if the model was a good fit, including the Chi-square statistic, standardized root-mean-squre residual (SRMR ≤ 0.08), root-mean-squre error of approximation (RMSEA ≤ 0.08), goodness-of-fit index (GFI ≥ 0.09), comparative fit index (CFI ≥ 0.09) [51]. Third, we used the bootstrapping method to identify the mediating role. This method yielded a 95% deviation corrected confidence interval of 839 samples of the data. Finally, due to the imbalance of gender ratio in our samples (229 men and 610 women), we further adopted *T* test and cross-gender path analysis to explore gender differences. Gender invariance could examine not only individual paths but also indirect effects. Specifically, two models were established, one was to allow unconstrained paths, the other was to restrict the path coefficients of the two genders to be equal, error variances and path variances invariable basic parameters [52]. If the Chi-square difference test is significant, it indicates that the fit of a model in which all paths are allowed to vary across gender is significantly worse than the fit of a model with parameters constrained to be equal across men and women [53]. And the chi-square difference tested to examine whether indirect effects differ across gender [53].

## Results

### Correlation analysis of Big Five personality traits, aggression and envy

The results of correlation analysis indicated that all variables were significantly correlated. Except that openness to experience was not significantly correlated with aggression (*r* = − 0.05, *p* = 0.146) (see Table 1). Meanwhile, Pearson’s *r* of 0.10 is interpreted as small effect, 0.30 medium, and 0.50 large [54]. The results supported hypothesis 1, i.e., neuroticism was positively correlated with aggression; agreeableness was negatively correlated with aggression.

**Table 1:** Means, standard deviations, and bivariate correlations of study variables

Variables	1	2	3	4	5	6	7	8
1. Neuroticism	_							
2. Extraversion	− .33***	_						
3. Openness to experience	− .11***	.39***	_					
4. Agreeableness	− .19***	.17***	.12***	_				
5. Conscientiousness	− .36***	.27***	.32***	0.23***	_			
6. Aggression	.37***	− .09**	− .05	− .46***	− .17***	_		
7. Malicious envy	.34***	− .21***	− .15***	− .33***	− .18***	.51***	_	
8. Benign envy	− .15***	.23***	.23***	.25***	.31***	− .20***	− .29***	_
*M*	72.46	77.44	81.57	85.04	78.73	61.48	23.15	11.38
*SD*	10.99	8.10	8.35	7.86	8.49	14.42	3.68	4.60
Theoretical score range	61.47 ~ 83.45	69.34 ~ 85.54	73.22 ~ 89.92	77.18 ~ 92.90	70.24 ~ 87.22	47.06 ~ 75.90	19.47 ~ 26.83	6.78 ~ 15.98

*N* = 839. **p < .01, ***p < .001

### A path analysis on aggression of Big Five personality traits and envy

**Fig. 1:**
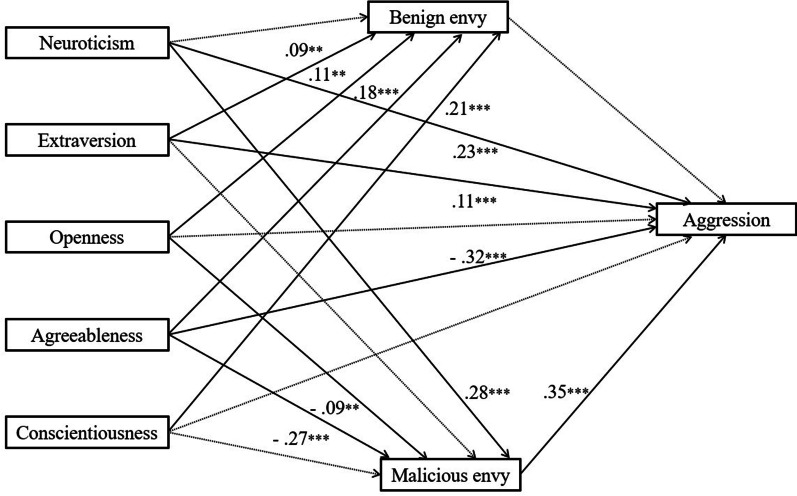
The solid line indicated statistically significant path, while the dotted line indicated that the path is not significant. *Note N* = 839. **p < .01, **p < .001

As it can be seen in Table 1, there was a significant correlation between variables. We then performed a path analysis to further explore the link between Big Five personality, aggression and benign/malicious envy. The results revealed that the model was just recognized, that is, the saturated model, and the fitting values were as follows: *χ*^2^ = 0, *df* = 0, RMSEA = 0.00, SRMR = 0.00; CFI = 1.00; GFI = 1.00. According to previous studies, the saturation model no longer focused on the fitting index, but only on whether its path coefficient was significant [55]. In return, some paths were not significant (e.g., Neuroticism → Benign envy, Extraversion → Malicious envy, Conscientiousness → Malicious envy, Openness to experience → Aggression, Conscientiousness → Aggression, Benign envy → Aggression). Therefore, set these paths to 0 [35] (see Fig. 1). The results showed the index as follows: *χ*^2^ = 4.00, *df* = 6, RMSEA = 0.00, SRMR = 0.01; CFI = 1.00; GFI = 1.00.

### Analysis on the mediating role of envy between the Big Five personality traits and aggression

Furthermore, a bootstrapping mediation analysis [56] was conducted to test the mediating role of envy between the Big Five personality traits and aggression. 2000 bootstrap samples (*N* = 839) were selected from the original data by random sampling. The results revealed that malicious envy plays a mediating role between neuroticism and aggression (*β* = 0.19, *p* < 0.001, 95% CI = [0.14, 0.24]). Meanwhile, malicious envy also played a mediating role between agreeableness and aggression equaled (*β* = − 0.24, *p* < 001, 95% CI = [− 0.32, − 0.17]) (see Table 2). Table 2 included path estimates (total, total indirect, specific indirect effects, with estimates and 95% CIs).

**Table 2:** Mediating effect of malicious envy between neuroticism/agreeability and progression and 95% confidence intervals

	Paths	Effect	Boot *SE*	Lower	Upper
Direct effect	Neuroticism → Aggression	.32	.04	.24	.39
Indirect effect	Neuroticism → Malicious envy → Aggression	.19	.03	.14	.24
Total indirect effect		.19			
Total effect		.51			
Direct effect	Agreeableness → Aggression	− .58	− .05	− .69	− .48
Indirect effect	Agreeableness → Malicious envy → Aggression	− .24	− .04	− .32	− .17
Total indirect effect		− .24			
Total effect		− .82			

### Gender difference

Due to the gender imbalance in the sample of this study, we first tested the gender differences of the Big Five personality traits, aggression and benign/malicious envy using the independent sample *T*-test. The results indicated that there were significant gender differences in neuroticism (*t*_(839)_ =  − 2.00*, p* = 0.046, cohen’s *d* = − 0.15*; M*_men_ ± *SD* = 71.23 ± 11.62, *M*_women_ ± *SD* = 72.93 ± 10.72), agreeableness (*t*_(839*)*_ = − 2.56; *p* = 0.011, cohen’s *d* = − 0.20; *M*_men_ ± *SD* = 83.91 ± 7.91, *M*_women_ ± *SD* = 85.46 ± 7.81*),* aggression (*t*_(839)_ = 3.44; *p* = 0.001*,* cohen’s *d* = 0.26; *M*_men_ ± *SD* = 64.25 ± 15.28, *M *_*women*_ ± *SD* = 6*0.43* ± *13.96*) and benign envy (*t*_(839)_ =  − 3.43, *p* = 0.001, cohen’s* d* = − 0.26; *M*_men_ ± *SD* = 22.44 ± 4.06;* M*_women_ ± *SD* = 23.41 ± 3.49). Gender differences in extraversion (*t*_(839)_ =  − 1.06, *p* = 0.291, cohen’s *d* = − 0.08), openness to experience (*t*_(839)_ = 0.36,* p* = 0.721, cohen’s *d* = 0.03), conscientiousness (*t*_(839)_ = 1.38,* p* = 0.167, cohen’s *d* = 0.11) and malicious envy (*t*_(839)_ = 0.87, *p* = 0.385, cohen’s *d* = 0.06*)* were not significant.

Then, the gender differences in the path were further explored by cross-gender path analysis. The results found that there was a significant difference [Δ *χ* ^2^ = 22.26, *df* = 4, *p* < 0.001]. Table 3 shown that all the fitting indicators of the two models reach the fitting standard. Meanwhile, this result revealed that the mediating role of malicious envy among extraversion, agreeableness and aggression was still significant in both men (*β* extraversion = − 0.14, *p* = 0.001, 95% CI = [0.07, 0.26]; *β* agreeableness = − 0.11, *p* = 0.012, 95% CI = [− 0.25, − 0.02]) and women (*β* extraversion = 0.12, *p* = 0.001, 95% CI = [0.08, 0.17]; *β* agreeableness = − 0.19, *p* = 0.001, 95% CI = [− 0.26, − 0.13]).

**Table 3:** Comparison of unconstrained and constrained path models

Paths	*χ*^2^	*df*	SRMR	RMSEA	CFI	GFI
Unconstrained paths	5.93	6	.02	.00	1.00	1.00
constraint paths	51.38	36	.04	.02	.99	.98

Moreover, critical ratios for differences (CRD, absolute value range > 1.96) was used as an indicator to further explore the cross-gender stability of pathways [57]. The results found that there were no significant differences in the paths of all variables (CRD _Neuroticism → Aggression_ = 1.50, CRD _Neuroticism → Malicious envy_ =—1.17, CRD _Extraversion → Benign envy_ = 1.08, CRD _Extraversion → Aggression_ = − 0.40, CRD _Openness to experience → Benign envy_ = − 0.20, CRD _Agreeableness → Benign envy_ = − 0.44, CRD _Agreeableness → Malicious envy_ = − 1.35, CRD _Agreeableness → Aggression_ = 1.73, CRD _Conscientiousness → Benign envy_ = 0.39, CRD _Malicious envy → Aggression_ = 0.42, CRD _Openness to experience → Malicious envy_ = − 0.57).

## Discussion

Based on the GAM, the current study examined the association between Big Five personality traits and aggression and the mediating role of benign/malicious envy. The results suggested that there was a positive correlation between neuroticism and aggression, and a negative correlation between agreeableness and aggression. Malicious envy not only plays a mediating role between neuroticism and aggression, but also between agreeableness and aggression. Meanwhile, the mediating role of malicious envy was also cross-gender stable. These results have important value for the understanding of the connection between personality and aggression.

### The link between the Big Five personality traits and aggression

Regression analysis revealed that neuroticism and extraversion were positively associated with aggression, agreeableness was negatively related to aggression, while openness to experience and conscientiousness were unrelated to aggression. First, this result is consistent with the GAM, and peoples with different personality traits also have aggressive behaviors [1]. Second, prior research has found agreeableness was negatively linked with aggression [58, 59], neuroticism and extraversion are positively related to aggression [18], openness to experience intends to be unrelated to aggression [17]. Thus, our study repeated previous findings.

To be specific, neuroticism was significantly associated with aggression. Those with higher levels of neuroticism were considered to be more prone to get easily upset. These individuals are thought to less stable emotions [60]. Thus, people that display many neurotic personality traits are more predisposed to emotional instability and more susceptible to conflict with others. On the contrary, agreeableness and aggression were consistently negatively associated [61]. Agreeableness, which was characterized as cooperative and understanding [62], is a facet that is related to the motivation to maintain positive interpersonal relationships [17]. Similarly, the relationship between agreeableness and mind indicates that the former is responsible for processing social information. Furthermore, agreeableness supports altruism. While aggression is a kind of destructive and hostile behavior that has anti-social tendencies [1]. Therefore, this may further explain the evidence we found that agreeableness negatively linked with aggression and the results of the study prove hypothesis 1.

In addition, the difference between this study and previous researches is no link between conscientiousness and aggression. Conscientiousness has also been called conformity or dependability [63]. There are differences in interpretation of the dimension of conscientiousness [64]. On the one hand, conscientiousness was considered to be dependability [60], on the other hand, Chinese people generally advocate collectivist culture [65], emphasizing social harmony and avoiding conflicts [66], so under this background, conscientiousness represents the obligation to perform as a group member and even make sacrifices. This cultural difference may lead to the irrelevance of conscientiousness for aggression of Chinese participants.

### The mediating role of malicious envy both in the relationship of neuroticism-aggression and agreeableness-aggression

In the following mediation analysis, we found that malicious envy mediated between neuroticism and aggression, while benign envy did not. Lange et al. [24] proposed the dual envy theory to explain this. The theory mentions that the components of malicious envy involve directed aggression, and non-directed aggression, whereas benign envy did not. People with higher levels of neuroticism and more frequent upward social comparisons are tend to experience hostile and resentful emotions, that is, malicious envy, thereby making it easier for them to make aggressive behaviors. Meanwhile, previous research has revealed that envy was a kind of emotion based on social comparison [37], and malicious envy is more of a hostile emotion, which tends to pull down envied people from their high positions. While benign envy tends to be a frustrating emotion, it is more likely to make one improve themself [23, 26]. People with high neurotic personality traits are more likely to experience malicious envy, which is a threat from others and a desire to bring the person envied down from high positions, and thus are associated with aggression.

Similarly, we found that malicious envy played a mediating role between agreeableness and aggression, but benign envy did not play said mediating role. In the context of upward social comparison, people with higher levels of agreeableness are less likely to experience feelings of malicious envy. Hence, then they are less likely to commit aggressive actions. In addition, people with agreeableness personality traits are cooperative and good at understanding [62], therefore, they will look at things from a good-natured perspective. In the social comparison of people, they will reduce the generation of hostility towards others, but they will appreciate the advantages of others, that is, they will reduce the aggressive behavior by reducing malicious envy. The results of the study support hypothesis two. However, the result differed from our hypothesis two in that benign envy did not play a mediating role in agreeableness and aggression. One possible explanation is that the motives of benign/malicious envy are different, i.e., the main motive of malicious envy is to attack others, while the main motive of benign envy is to improve oneself [67].

### Gender difference

Consistent with previous studies, women scored higher than men in both agreeableness and neuroticism [68]. The *evolutionary* explanation is that women are more agreeable and neuroticism because, in previous eras, such behavior was beneficial to child survival and, in turn, had an evolutionary advantage over other traits associated with it [68]. Additionally, consistent with previous research, men have higher aggression than women [69–72]. Social role theory suggests that men are more aggressive than women because male gender roles allow aggression while female gender roles prevent it [73]. However, women have higher levels of benign envy than men. This is supports by recently published research [41] that girls are less likely to experience feelings of hostility and depression and more likely to experience benign envy during upward comparison. However, cross-gender path analysis found that there is no gender difference in the above variables. Future research can further explore the path differences among personality, envy and aggression. Meanwhile, in both men and women, the mediating role of malicious envy between neuroticism, agreeableness and aggression is remarkable. This indicates that the mediating role of malicious envy is stable, and future research can further verify the role of malicious envy in the relationship between personality and aggression.

### Limitations and future directions

It is important to note that the participants were all Chinese college students. Future research will continue exploring the mechanisms of benign/malicious envy within the context of the Big Five personality traits and aggression from different cultures and groups. Second, this study used the questionnaire method that cannot show the causal connection between the Big Five personality traits and aggression. Therefore, future research could further explore the link between the Big Five personality traits and aggression in the laboratory context or using longitudinal study. Third, this study suggested the link between the Big Five personality traits and aggression from an envy’s affective perspective. Lange et al. [24] revealed that envy encompasses variety of diverse affective, cognitive, and motivational aspects. Future studies can further explore the mechanism from envy’ cognitive and motivational perspective. Finally, this paper did not measure the benign and malicious envy at the state level. It should be underlined that the types of state of envy generated by a specific episode depend on two appraisal dimensions: deservingness and personal control, and are not always related to one’s tendency of envy (trait-like) [24]. Therefore, future research could consider exploring the mechanism of action between the two types of envy at the state level between the Big Five personality traits and aggression.

## Conclusion

Based on the GAM, the current study first to revealed the link between the Big Five personality traits and aggression. Therefore, this contributes to the personality base of envy, especially benign/malicious envy, which fills the blank of personality and benign/malicious envy. More importantly, we have discovered the mediating role of envy. As a side of envy, malicious envy plays an important mediating role in the influence of these two personality traits on aggression, and the mediating role of malicious envy has cross-gender stability. The current study not only extended the GAM, but also further revealed the relationship between personality, especially neuroticism and agreeable, and aggression, and the role of benign/malicious envy in it.

## Data Availability

The datasets generated and/or analysed during the current study are not publicly available due to this article has not been published but are available from the corresponding author on reasonable request.

## Abbreviations

GAM: The general aggression model
SRMR: Standardized root-mean-squre residual
RMSEA: Root-mean-squre error of approximation
GFI: Goodness-of-fit index
CFI: Comparative fit index
ECVI: Expected cross-validation index
